# eDNA Metabarcoding- and Microscopic Analysis for Diet Determination in Waterfowl, a Comparative Study in Vejlerne, Denmark

**DOI:** 10.3390/biology12091272

**Published:** 2023-09-21

**Authors:** Anna-Sofie Lützhøft Svendsen, Louise Bach Nielsen, Jakob Braüner Schmidt, Dan Bruhn, Line Holm Andersen, Cino Pertoldi

**Affiliations:** 1 Department of Chemistry and Bioscience, Aalborg University, Fredrik Bajers Vej 7H, DK-9220 Aalborg Øst, Denmark; bach.n@rn.dk (L.B.N.); 302762@viauc.dk (J.B.S.); db@bio.aau.dk (D.B.); lihoan@bio.aau.dk (L.H.A.); cp@bio.aau.dk (C.P.); 2 Department of Zoology, Aalborg Zoo, Mølleparkvej 63, DK-9000 Aalborg, Denmark

**Keywords:** eDNA metabarcoding, microscopy, waterfowl, diet determination, conservation biology, frequency of occurrence, cumulative curves, taxonomic resolution

## Abstract

**Simple Summary:**

eDNA metabarcoding is a relatively novel method for studying the diet of wild animals. This study compares eDNA metabarcoding with microscopy, in order to determine the diet of four species of waterfowl. In total, 56 plants were identified at the species level. The study concluded that the combination of morphological analysis and DNA metabarcoding can yield adequate information to address pertinent ecological inquiries.

**Abstract:**

Understanding diets and structural food webs are keys to the apprehension of ecological communities, upon which conservation and management biology are based. The understanding of grazing and habitat choice for waterfowl is one of the most important topics for avian ecologists today and can, to some degree, be answered by dietary analysis. Droppings collected from four waterfowl, the Eurasian wigeon (*Anas penelope*), Greylag goose (*Anser anser*), pink-footed goose (*Anser brachyrhynchus*) and Barnacle goose (*Branta leucopsis*) in Vejlerne (Denmark), were analysed microscopically and through eDNA metabarcoding with the use of next generation sequencing (NGS) to accumulate knowledge about the diet of these waterfowl. In total, 120 dropping samples were microscopically analysed, of which the eDNA metabarcoding analysis was done on 79 samples. The prey items were identified according to the taxonomic level of species, and a qualitative method, frequency of occurrence (FO) and FO calculated as a percentage, was used in order to compare the results from the two methods. As neither of the methods was able to encompass all species discovered when combining the two methods, it was concluded in this study that the two methods can support each other in a dietary analysis of waterfowl, but not replace one another.

## 1. Introduction

With just 5% of the terrestrial surface of Earth (excluding Antarctica) left untouched by man [1], most areas of conservation concern are subject to human management to some extent [2]. Therefore, management biology is essential to the conservation of areas and species. Information and understanding of the ecological community, its energy flow, as well as intra- and interspecific interactions are necessary for proper management, as this information is a requisite for proper management. Knowledge about structural food webs and interactions within and between trophic layers is part of understanding the ecological community [3,4,5,6,7,8,9,10] and if the main conservation interest is a management plan for specific animals, the basis for these plans can be enlightened through dietary analysis [3].

Waterfowl are considered the dominant fauna in the Northern Hemisphere and have long been of vast importance in the studies of avian ecologists [11]. For a considerable period of time, there was little to no focus on waterfowl habitats between breeding seasons. This focus has shifted, and habitat choice, studied, e.g., by estimating an area’s capacity, hydrological importance, and resources availability, is considered to be one of the most important questions for avian ecologists to answer today [11]. Dietary analysis is the foundation for waterfowl ecology, as an understanding of consumed prey items can help shed light on food availability and expand the understanding of potential food preferences and niche overlaps [7]. An argument could be made for in situ observation of consumed prey items, as this potentially increases accuracy, though this would be abundantly time and money-consuming [3]. The preferred method for conducting dietary analyses is by euthanizing avian wildlife and conducting an invasive sampling [12]. Further, these studies often use frequency of occurrence (FO) and weight of samples, as it has been shown to be impossible to produce reliable results through the use of quantitative methods, because it is very difficult to identify every fragment of the ingested food items of herbivores [12,13,14]. The analysis of faecal samples is a viable alternative to invasive sampling and is preferable when studying endangered species, where one might not want to or have the option to euthanize the animals in question [3,13]. Faecal samples from herbivorous and carnivorous animals can be analysed both microscopically and by using environmental DNA (eDNA) [6,7,15,16]. However, researchers focusing on faecal samples for their dietary investigation should consider the obstacles this method provides. For herbivorous birds, some of the caveats of microscopic faecal dietary analysis are if the number of different plants consumed is too high or the samples are merely too degraded; here, it might be impossible to differentiate plant material to a taxonomically lower level than family [13]. The level of precision is of course also affected if using degraded samples, when considering using next-generation sequencing (NGS) for eDNA analysis [5,7]. Next-generation sequencing is applied during the metabarcoding of eDNA, which is a process that can shotgun multiple taxa simultaneously, resulting in a comprehensive list of what species the environmental sample might contain [17].

The aim of this study is to compare two non-invasive methods to determine the dietary needs of herbivorous birds: eDNA metabarcoding and microscopic analysis. The study conducted for the article utilised droppings collected from four herbivorous waterfowl: the Eurasian wigeon—*Anas penelope*, Greylag goose—*Anseranser*, pink-footed goose—*Anser brachyrhynchus*, and Barnacle goose—*Branta leucopsis*. The four species are on the IUCN red list as least concern [18,19,20,21], and the populations of *A. anser*, *A. brachyrhynchus* and *B. leucopsis* are all increasing [19,20,21]. The population of *A. penelope* in Northwestern Europe is, however, contemporarily declining [22]. The four species occur in high numbers in the Danish scientific reserve Vejlerne [23], in which this study was conducted. Many different plant species can be found on the salt marsh and in the reed beds in Vejlerne [24,25,26]. By combining microscopic analysis with DNA barcoding on the undigested and undegraded plant material in the faecal samples, we investigated the capabilities of the two different methods to answer the questions, ‘Which plants do the four different species feed upon, and is there a food overlap among the species?’

## 2. Materials and Methods

### 2.1. Study Location

**Figure A1:**
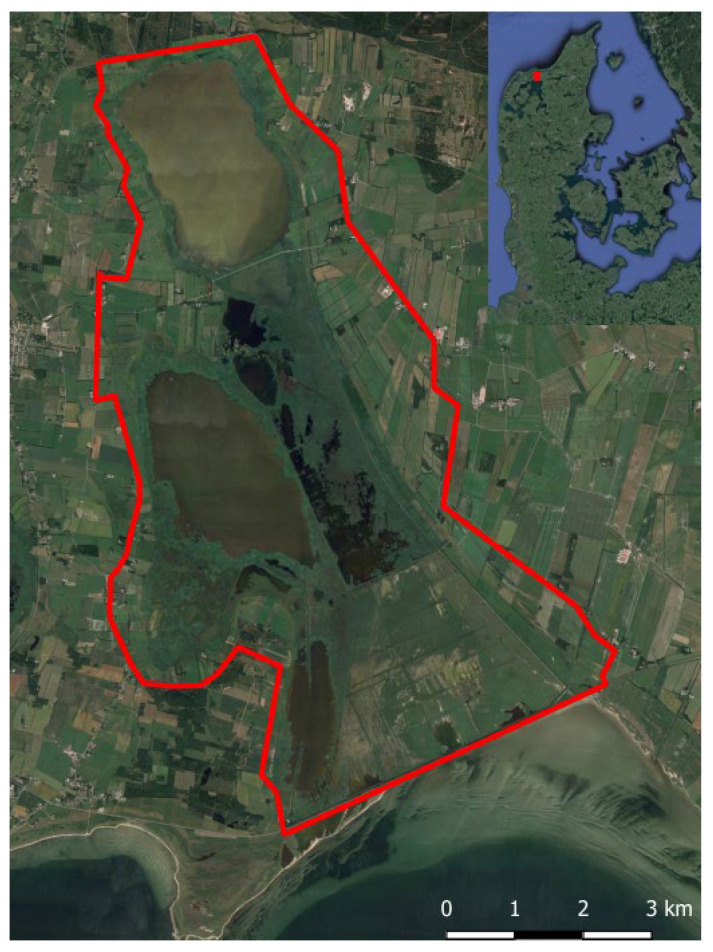
Area map of the eastern part of Vejlerne, Denmark, where samples used for the study, described in this article, were collected (QGIS Development Team [49]).

The droppings were collected in the eastern part of Vejlerne nature reserve (Figure A1). Vejlerne is the biggest ornithological sanctuary in Northern Europe and is protected under several regulatory guidelines [27]. The reserve consists of marshes, low freshwater, and brackish lakes and also contains the largest continuous reed bed in Scandinavia [28]. More than 300 bird species have been registered in Vejlerne, of which at least 130 are breeding in the area [27]. The populations of *A. brachyrhynchus* and *B. leucopsis* have been increasing in the area, while the populations of *A. anser* and *A. penelope* have been stable [29]. Vejlerne is a stopover site for the four bird species, on their migration to Southern Europe for the winter [27]. They arrive in September, and in mild winters some might stay during the winter [30].

### 2.2. Samples and Preparations

This study is based on 120 faecal samples from four waterfowl species: *A. penelope*, *A. anser*, *A. brachyrhynchus* and *B. leucopsis*. These samples were collected in the months of October and November of 2017, in the eastern part of the natural reserve Vejlerne. Fresh droppings were collected, by observing flocks of each separate species foraging, and collecting fresh droppings when the flocks left the area. The samples were subsequently stored at −20 °C [15]. In the laboratory, all samples were divided into two subsamples: one part for microscopic analysis and one part for eDNA analysis. Subsamples for microscopic analysis were prepared in individual Petri dishes along with water and soap to help dissolve the droppings and better access the pieces of plant epidermis [10,31] and kept refrigerated at 5 °C until analysis, while subsamples for eDNA metabarcoding were stored in 1.5 mL eppendorf tubes at −20 °C [15].

We compiled a reference list of potential food items with pictures of plant epidermal layers [32] by first collecting fresh plant material in Vejlerne and from any surrounding agricultural landscape. A total of 22 candidate species were collected, including some crops as well as the aquatic species *Zostera marina* (Table A1) [33,34]. Next, each of these species was taped to a piece of paper and covered lightly with several independent spots of nail polish. After a period of drying, the nail polish was removed, fitted to microscope slides, and put under a microscope. This allowed pictures to be taken of the abaxial epidermal layer for each of the 22 plant species, thereby allowing comparison to epidermal structures found in the faecal subsamples [32].

### 2.3. Microscopic Analysis

To analyse the subsamples microscopically, each subsample was stirred in its petri dish for 30 s, and a minor amount of the subsample was added to a microscope slide and spread out evenly to cover approximately ⅓ of the slide. The first 10 observed pieces of epidermis were determined to the taxonomic level of species. The determination of the species was based on the morphological characteristics of the epidermis in comparison to the reference picture list of the 22 plant species (Table A1).

A total of 120 subsamples were analysed microscopically: 55 subsamples from *A. penelope*, 25 subsamples from *B. leucopsis*, and 20 subsamples from each of the two species *A. anser* and *A. brachyrhynchus.* A Carl Zeiss Axiolabdrb KT 450,905 trinocular 12 fluorescence microscope was used. FO (the number of faecal samples in which a species was detected) was stated, and the corresponding percent (%) was calculated as the number of faecal samples in which a species was detected divided by the total number of detections times 100, similar to Pertoldi et al. 2021 [10].

### 2.4. DNA Extraction

The subsamples for eDNA analysis were extracted using the DNeasy Blood and tissue Kit (Qiagen, Hilden, Germany), following the manufacturer’s protocol. The study resulted in the extraction of 79 subsamples for eDNA metabarcoding: 21 subsamples from *A. penelope*, 20 subsamples from *B. leucopsis*, and 19 subsamples from each of the other two birds, *A. anser* and *A. brachyrhynchus.*

### 2.5. PCR

Amplicon libraries for the trnL gene (chloroplast) were prepared by a custom protocol based on an Illumina protocol [35]. Up to 10 ng of extracted DNA were used as a template for PCR amplification. Each PCR reaction (25 μL) contained (12.5 μL) PCRBIO Ultra mix and 400 nM of each forward and reverse tailed primer mix. PCR was done with the following programme: initial denaturation at 95 °C for 2 min, 30 cycles of amplification (95 °C for 15 s, 50 °C for 15 s, 72 °C for 50 s) and a final elongation at 72 °C for 5 min. Duplicate PCR reactions were performed for each sample and the duplicates were pooled after PCR. The forward and reverse, tailed primers were designed according to Nierychlo et al. 2020 [35] and contain primers targeting the trnL gene [trnL c] CGAAATCGGTAGACGCTACG and [trnL d] GGGGATAGAGGGACTTGAAC [36]. The primer tails enable the attachment of Illumina Nextera adaptors necessary for sequencing in a subsequent PCR. The resulting amplicon libraries were purified using the standard protocol for CleanNGS SPRI beads (CleanNA, Waddinxveen, The Netherlands) with a bead-to-sample ratio of 4:5. The DNA was eluted in 25 μL of nuclease free-water (Qiagen, Germany). DNA concentration was measured using the Qubit dsDNA HS Assay kit (Thermo Fisher Scientific, Waltham, MA, USA). Gel electrophoresis using Tapestation 2200 and D1000/High sensitivity D1000 screentapes (Agilent, Santa Clara, CA, USA) was used to validate product size and purity of a subset of amplicon libraries. Sequencing libraries were prepared from the purified amplicon libraries using a second PCR. Each PCR reaction (25 μL) contained PCRBIO HiFi buffer (1×), PCRBIO HiFi Polymerase (1 U/reaction) (PCR Biosystems Ltd., London, UK), adaptor mix (400 nM of each forward and reverse) and up to 10 ng of amplicon library template. PCR was done with the following programme: initial denaturation at 95 °C for 2 min, 8 cycles of amplification (95 °C for 20 s, 55 °C for 30 s, 72 °C for 60 s) and a final elongation at 72 °C for 5 min. The resulting sequencing libraries were purified using the standard protocol for CleanNGS SPRI beads with a bead-to-sample ratio of 4:5. DNA was eluted in 25 μL of nuclease-free water. DNA concentration was measured using the Qubit dsDNA HS Assay kit. Gel electrophoresis using Tapestation2200 and D1000/High sensitivity D1000 screentapes was used to validate product size and purity of a subset of sequencing libraries.

### 2.6. Sequence Analysis and Filtering

The purified sequencing libraries were pooled in equimolar concentrations and diluted to 2 nM. The samples were paired-end sequenced (2 × 300 bp) on a MiSeq (Illumina, San Diego, CA, USA) using a MiSeq Reagent kit v3 (Illumina, San Diego, CA, USA) following the standard guidelines for preparing and loading samples on the MiSeq. A total of > 10% of the PhiX control library was spiked in to overcome low-complexity issues often observed with amplicon samples. The resulting sequence reads were imported into MEGA 7.0.26 [37] for analysis in BLAST https://blast.ncbi.nlm.nih.gov/Blast.cgi (accessed on 10 May 2018). De novo with ≤9 reads in a sample were not included, and species of algae were not considered in this study.

### 2.7. Cumulative Curves

By using Vegan-package—2.5-1 [38] in RStudio version—3.4.3 [39] species accumulation curves were calculated for the dietary richness at the four taxonomic levels: order, family, genus and species. This was done both for the subsamples that were analysed by microscopy and for the subsamples that were analysed by eDNA metabarcoding. A vertical limit illustrating a 95% confidence interval was fitted in each figure. Horizontal lines, colour corresponding to the type of analysis, were added to illustrate the theoretical maximum richness saturation possible, which was calculated as Chao estimates [38].

## 3. Results

### 3.1. Results of Microscopic Analysis

Across all 120 subsamples analysed by microscopy, a total of 19 plant species were detected (Table 1). Some species have only been found in one or very few subsamples, while others have been found in a higher number of subsamples. For *A. penelope*, 14 plant species were detected, and the highest FO was for *Glaux maritima*, *Agrostis stolonifera* and *Festuca rubra*. For *A. anser* 13 species were detected, and the highest FO was for *Holcus lanatus*, *Glaux maritima* and *Festuca rubra*. For *A. brachyrhynchus*, 13 species were detected, and the highest FO was for *Holcus lanatus*, *Agrostis stolonifera* and *Festuca rubra*. For *B. leucopsis*, 12 species were detected, and the highest FO was for *Holcus lanatus*, *Festuca rubra* and *Agrostis stolonifera*. The aquatic plant *Zostera marina* was detected for *A. penelope* and the crop *Triticum aestivum* was detected for *A. penelope* and *A. brachyrhynchus* and *Hordeum vulgare* was detected for *A. anser*.

### 3.2. Results of eDNA Metabarcoding

Across all 79 analysed subsamples analysed by eDNA metabarcoding, a total of 612,057 reads were obtained (Table A2). Of that, >70% were reads that pertain to plants. A total of 49 plants were detected at the species level across the subsamples for all four waterfowl species. Some species have only been found in one or very few subsamples, while others have been found in a higher number of subsamples (Table 2). The species found in the most subsamples from *A. penelope* were *Poa trivialis*, *Potentilla anserina* and *Juncus gerardii*, while a total of 24 plants were determined to species level. The number of plants determined to species level for *A. anser* was 34, and the species found in the most subsamples were *Poa trivialis*, *Potentilla anserina* and *Juncus gerardii*. The species found in the most subsamples from *A. brachyrhynchus* were *Festuca arundinacea*, *Poa trivialis* and *Alopecurus geniculatus*, while a total of 35 plants were determined to species level. The number of plants determined to species level for *B. leucopsis* was 29, and the species found in the most subsamples were *Festuca arundinacea*, *Alopecurus geniculatus* and *Poa trivialis*. The aquatic plants *Batrachium spp.*, *Utricularia australis* and *Potamogeton perfoliatus* were detected in samples from *A. penelope*, and *Potamogeton perfoliatus* was also detected in samples from *A. anser*. The crop plants *Triticum aestivum* and *Hordeum vulgare* were detected in subsamples from *A. anser* and *A. brachyrhynchus*, while the crop *Brassica napus* was detected in samples from *A. anser* and *B. leucopsis*.

### 3.3. Presence/Absence of Plant Species by Each Method

Taking both methods into account, a total of 56 plants were determined to species level in the droppings analysed in this study (Table 3). Of the 56 plant species, 12 were found both by microscopic analysis and eDNA metabarcoding in droppings from some of the birds (Table 3). These include *Agrostis capillaris*, *Potentilla anserina*, *Cynosurus cristatus*, *Glaux maritima*, *Holcus lanatus*, *Hordeum vulgare*, *Leontodon autumnalis*, *Phragmites australis*, *Plantago maritima*, *Trifolium pratense*, *Triglochin maritima* and *Triticum aestivum*. Some of the species found by eDNA metabarcoding (Table 2) were not on our list of candidate species (Table A1), but they have been found in Vejlerne. These include *Bellis perennis*, *Cerastium fontanum*, *Eleocharis uniglumis*, *Juncus gerardii*, *Lychnis flos-cuculi*, *Plantago major*, *Poa pratensis*, *Poa trivialis*, *Ranunculus repens*, and *Spergularia media*, *Trifolium repens*, *Schoenoplectus tabernaemontani*, as well as a species within the genus *Anthoxanthum*, *Cirsium*, *Galium*, and *Sagina* [26]. Furthermore, some species have not been detected in Vejlerne, but are found in wet meadows and salt marshes throughout Northern Jutland. These include *Alopecurus geniculatus*, *Juncus bufonius*, *Juncus conglomeratus*, *Rumex crispus*, and *Vicia sativa*, as well as species of the genus *Achillea*, *Briza* [26] and *Kindbergia praelonga* [40]. Some species have not been detected in Vejlerne, but other species within the same genus have. This is true for the following species identified by eDNA: *Carex lasiocarpa*, *Festuca arundinacea*, *Juncus bulbosus* and *Poa supina* [26]. Other species identified by eDNA are not detected in Vejlerne, but species within the same genus have been found in wet meadows and salt marshes in Denmark. These include *Calamagrostis arundinacea*, *Cardamine hirsuta*, *Cardamine impatiens*, *Glyceria declinata* and *Myosotis arvensis* [26]. Some species have not been detected in Vejlerne but are common throughout Jutland: *Pinus mugo* [40], *Brassica napus*, *Betula pendula*, *Catabrosa aquatica* [41], and species within the genus *Solanum* [41] as well as species within *Batrachium* [42]. Some rare genera and species were also detected by eDNA metabarcoding. The following are either rare in Jutland or in Denmark in general: *Anisantha sterilis* [43], *Cuscuta* spp., *Meconopsis* spp., *Myosurus minimus*, *Potamogeton perfoliatus*, *Schoenus nigricans*, *Utricularia australis* [40], and *Drepanocladus sendtneri* [44].

### 3.4. Observed Richness and Chao Estimates

The observed richness for the subsamples from *A. penelope* analysed by microscopy resulted in a complete overlap between order and family, and a complete overlap between genus and species (Table 4). The same is true between order and family for the other three species of waterfowl. For the subsamples analysed by eDNA metabarcoding, there was no overlap between either of the levels for any of the waterfowl species. The cumulative number of taxa in the samples did not reach the theoretical maximum, the calculated Chao estimate (Table 4).

### 3.5. Cumulative Curves Results

**Figure 1:**
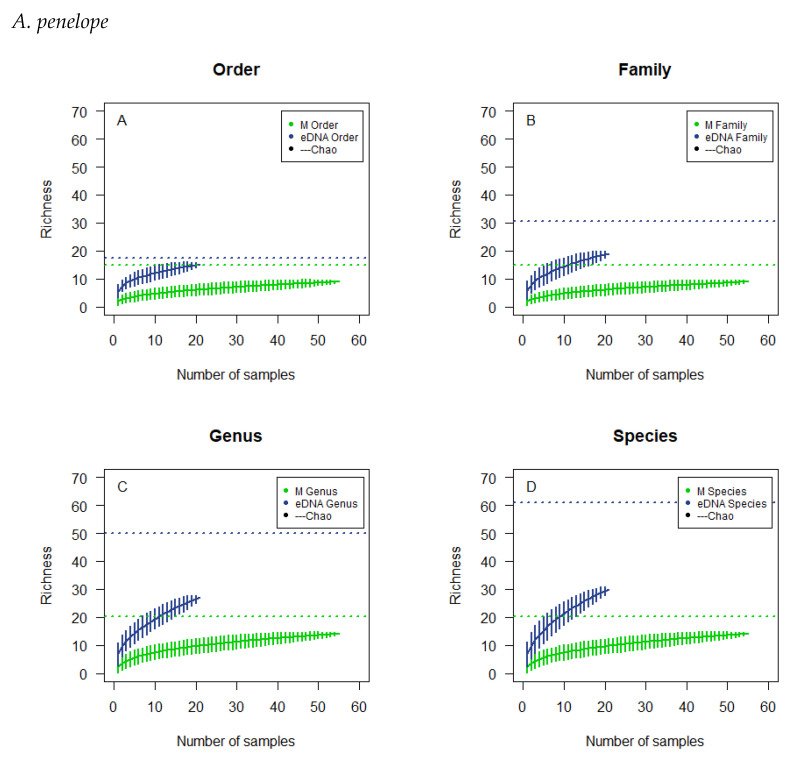
Cumulative curves and Chao estimated the richness of the microscopic analysis and the eDNA analysis of the droppings of *A. penelope*. The cumulative analyses were at the order level (**A**), family level (**B**), genus level (**C**), and species level (**D**). Horizontal, dotted lines, colour corresponding to the type of analysis, illustrate the chao estimates.

The cumulative curves for *A. penelope* are based on 55 and 21 subsamples for microscopy and eDNA metabarcoding, respectively (Figure 1). For the microscopically analysed samples the curves seem to flatten out somewhat early on the graph at all four taxonomic levels, though order and family reach a flattened plateau at a lower richness than genus and species. For the samples analysed by eDNA metabarcoding, the curves reach a higher richness than for microscopy, and they do not tend to flatten to a particular degree. The 95% confidence interval fitted on the resulting line for each taxonomic level is rather large for both methods. None of the curves reach the estimated richness.

**Figure 2:**
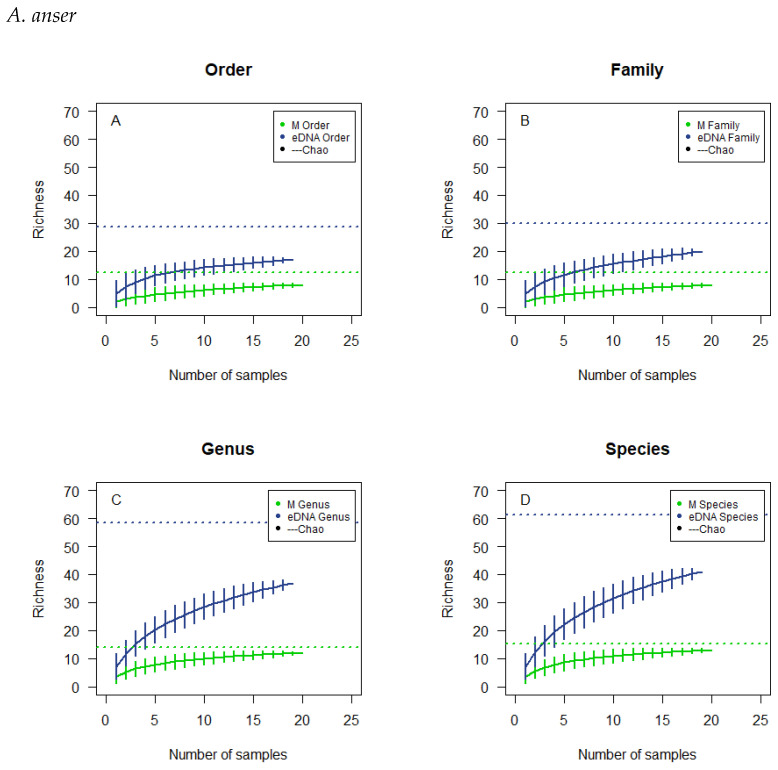
Cumulative curves and Chao estimated the richness of the microscopic analysis and the eDNA analysis of the droppings of *A. anser*. The cumulative analyses were at the order level (**A**), family level (**B**), genus level (**C**), and species level (**D**). Horizontal, dotted lines, colour corresponding to the type of analysis, illustrate the chao estimates.

The cumulative curves for *A. anser* are based on 20 and 19 subsamples for microscopy and eDNA metabarcoding respectively (Figure 2). For the microscopically analysed samples a flattened plateau is reached more expeditiously at the taxonomic levels of order and family and at a lower richness than genus and species. For the samples analysed by eDNA metabarcoding, the curves reach a higher richness than the curves for microscopy, and they do not tend to flatten to a particular degree. The 95% confidence interval fitted on the resulting line for each taxonomic level is rather large for both methods. None of the curves reach the estimated richness.

**Figure 3:**
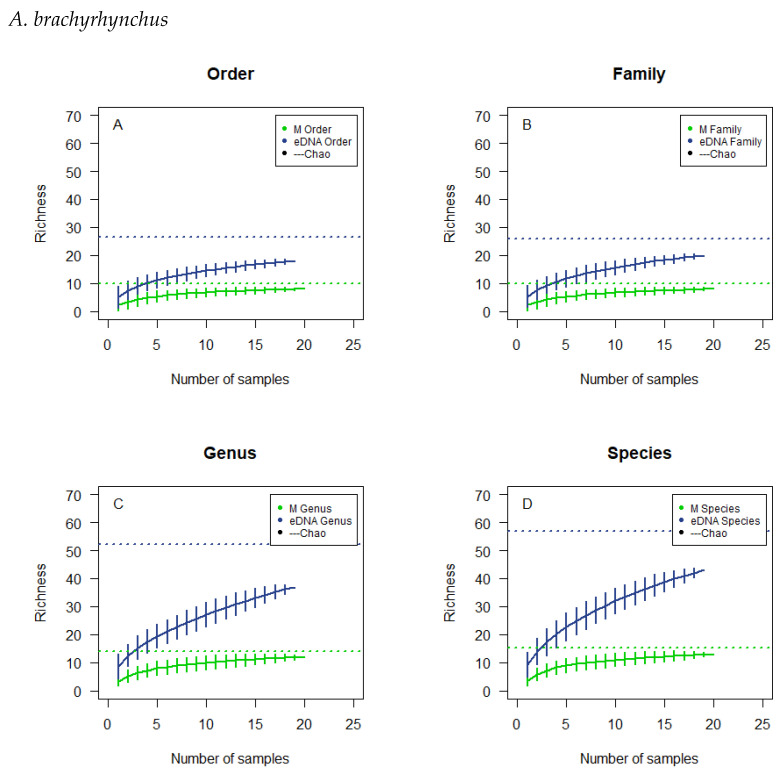
Cumulative curves and Chao estimated richness of the microscopic analysis and the eDNA analysis of the droppings of *A. brachyrhynchus*. The cumulative analyses were at the order level (**A**), family level (**B**) genus level (**C**) and species level (**D**). Horizontal, dotted lines, colour corresponding to the type of analysis, illustrate the chao estimates.

The cumulative curves for *A. brachyrhynchus* are based on 20 and 19 subsamples for microscopy and eDNA metabarcoding respectively (Figure 3). For the microscopically analysed samples a flattened plateau is reached more expeditiously at the taxonomic levels of order and family and at a lower richness than genus and species. For the samples analysed by eDNA metabarcoding the curves reach a higher richness than the curves for microscopy, and they do not tend to flatten to a particular degree. The 95% confidence interval fitted on the resulting line for each taxonomic level is rather large for both methods. None of the curves reach the estimated richness.

**Figure 4:**
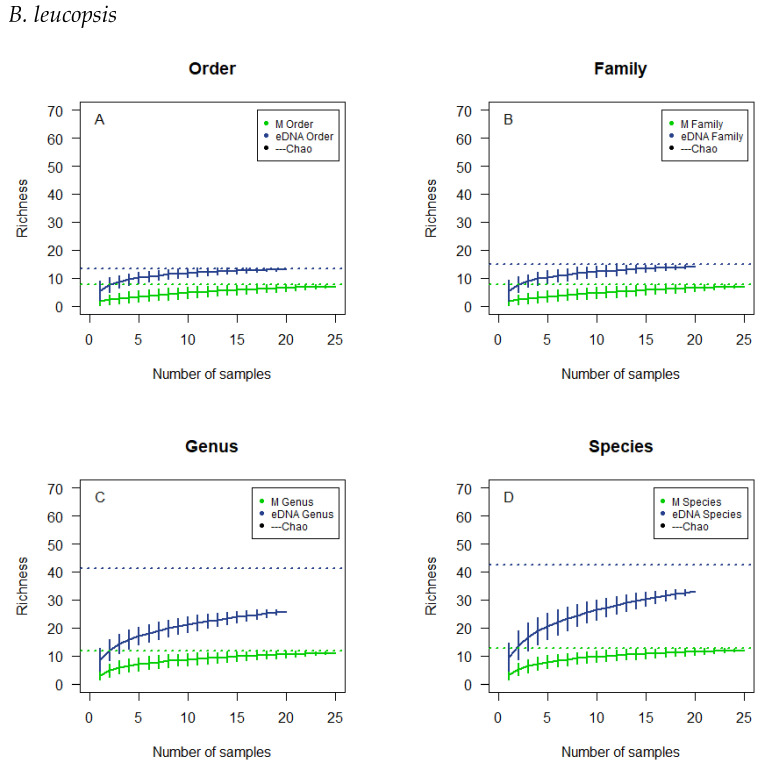
Cumulative curves and Chao estimated the richness of the microscopic analysis and the eDNA analysis of the droppings of *B. leucopsis*. The cumulative analyses were at the order level (**A**), family level (**B**), genus level (**C**), and species level (**D**). Horizontal, dotted lines, colour corresponding to the type of analysis, illustrate the chao estimates.

The cumulative curves for *A. brachyrhynchus* are based on 25 and 20 subsamples for microscopy and eDNA metabarcoding respectively (Figure 4). For the microscopically analysed samples a flattened plateau is reached more expeditiously at the taxonomic levels of order and family and at a lower richness than genus and species. For the samples analysed by eDNA metabarcoding the curves reach a higher richness than the curves for microscopy, and they do not tend to flatten to a particular degree. The 95%-confidence interval, fitted on the resulting line for each taxonomic level, is rather large for both methods. None of the curves reach the estimated richness.

## 4. Discussion

This study shows that combined, the two non-invasive methods of dietary analysis, microscopic and eDNA analysis of faecal samples, are capable of providing a good overview of the diet of herbivorous birds. However, our study also highlights the importance of using both methods in combination, as neither in itself gives a full overview of the diet. This has also been shown to be true between macroscopic and eDNA analysis on otter spraints [10]. The study of herbivore grazing patterns offers a multitude of possibilities when selecting methods for analysis [45]. This is however primarily when considering the choice of method in a study for collecting the datasets. The statistical methods of a study, however, will have a massive impact on the results one gathers and the questions examined [4]. For the study described in this article, only a qualitative method, FO, was applied. This was done as it has been shown numerous times that quantitative methods are unreliable for the study of bulk estimation of epidermal plant material [12,13,14]. When using a qualitative method, it will only be possible to distinguish whether or not a food item is present or absent in the sample, and not if it is a single or multiple occurrences [4]. This method may result in increased importance for rare or smaller food items as, if they are present even in small quantities, they will receive a value equal to that of food items that are massively present. In contrast, qualitative methods using FO can give insights into food items or categories that are not usually considered important, undeterred by the size of the minor bulk they might consist of [4]. It is therefore essential to understand what the given study is trying to answer and whether it is actually able to answer, considering the focus of the study and the material and methods available [4]. It is advantageous to some degree to conduct the microscopic analysis, because this does not require advanced laboratory work and is relatively simple to perform. However, to perform this satisfactorily and end up with correctly analysed epidermal plant items, to the taxonomical level of species, is so difficult that it is nearly impossible [6,13]. It should also be taken into account that epidermal plant identification is non-observational and contains intervariation amongst researchers resulting in biased decisions, which can lead the method to become biased [4]. This intervariation amongst researchers conducting similar investigations, and perhaps even with differentiating applications of the same or similar methods, might result in studies that are incomparable [4]. One of the limits for microscopic analysis is the degrading of samples, which primarily happens through the animals’ digestive system. The results of this can be that some plant species do not appear to have occurrence in the faeces [45,46]. Quantities of a species consumed, if not of considerable size, might be too small to be observed, resulting in negligence of the presence of rare taxa [4,45]. Any and all herbaceous elements consumed by herbivores result in fragments that can be found and identified through faecal analysis [45], though it can be difficult to distinguish species from the same genus from each other or even differentiate between genus in the same family, which is true for the two species *Festuca rubra* and *Festuca ovina* [13]. These issues could prove relevant for the study conducted for this paper.

Like the microscopic analyses, the use of eDNA metabarcoding analyses can provide useful insights as well as complications. The fact that both methods can be performed non-invasively is favourable to invasive sampling, which demands euthanised test subjects [47]. It is theorised that the use of metabarcoding can result in a higher taxonomic resolution than microscopic analysis [6]. This would in turn provide greater accuracy the understanding and analysis of herbivorous grazing patterns and interactions [6]. Using eDNA metabarcoding, it could prove advantageous to produce specific primers for a particular array of taxa that waterfowl eat. First, though, it would be necessary to assimilate a vast understanding of the ecological situation both the birds and plants in question are in. The result of this would, in theory, increase the overall accuracy of the investigation one would seek to commence [6].

DNA can result in a significantly higher FO than its macroscopic counterpart, meaning that it is more likely to detect consumed taxa by using DNA, and it has also been shown that DNA is more consistent in its presence in scat over time [3]. However, DNA in amounts not great enough might not be amplified enough; trace amounts <5% will result in amounts in too small quantities for further sequencing [4]. It should as well be remembered that the deterioration over time of DNA, when exposed to the surrounding environment, will happen at an increased rate compared to the harder parts of the droppings [47]. It could also be speculated, that species will be detected in the droppings, if the birds carelessly ingest plants, that are not food items, while the bird forages, or if the plants are ingested with the drinking water. The number of subsamples in which a certain plant species has been found might shed light on this specific issue. It is important to consider the plant species found by eDNA metabarcoding, and their presence in the habitat where the birds forage. Most of the plant species detected with this method in the present study have either been observed in Vejlerne, or are commonly found in wet meadows and salt marshes throughout Jutland. Some species have not been detected in Vejlerne, though species within the same genus have been detected either in Vejlerne or in wet meadows and salt marshes in Denmark. Most species on the list are therefore potential food items or closely related to food items. Considering the rare genera and species detected by eDNA metabarcoding, either the genus or family was likely to be present in the sampling area, suggesting that the lack of an appropriate reference in the sequence database might have led to the identification of an incorrect but closely related species.

Many of the plant species detected in the present study coincide with other studies on the diet of the given bird species, while some plant species are not considered part of the diet of the birds [34]. *A. brachyrhynchus* and *A. anser* are both earlier recorded foraging on waste grain in stubble fields, where they eat *Hordeum vulgare* and *Triticum aestivum* [34], which are also species detected in the present study. Clover has also been recorded for *A. brachyrhynchus* [34], of which *Trifolium repens* has been detected in the current study, and for *A. anser*, *Poa pratensis* and *Phragmites australis* were earlier recorded [34] and also detected in the current study. Plant species recorded in the diet of *B. leucopsis* and also detected in the present study consist of *Festuca* spp., *Poa* spp., *Agrostis* spp., *Puccinellia* spp., *Trifolium repens*, *Agrostis stolonifera*, *Puccinellia maritima*, *Festuca rubra*, and *Holcus lanatus* [34]. *A. penelope* has been recorded to eat *Zostera* spp. [34], which in the present study was only detected by microscopy. The diet of both *A. penelope* and *A. anser* is also recorded to consist of *Puccinellia maritima* [34], which was not detected in the present study. When attempting to analyse the diet of specific bird species, it is essential to keep in mind that food preferences are likely to differentiate depending on season and geographical regions, and that the diet will reflect availability and food preferences in the area of investigation [48]. Looking at the detected species by eDNA metabarcoding, a certain overlap in diet between the species of waterfowl could be suggested, as the species detected in most subsamples were widely the same across the four waterfowl species (Table 2). The same is true for the microscopic analysis, where the species with the highest FO were to a wide extent the same across the four waterfowl species (Table 1). Of the 12 plant species detected by both eDNA metabarcoding and microscopic analysis (Table 3), *Hordeum vulgare* and *Triticum aestivum* are crop species, which indicate that the waterfowl are not only foraging in Vejlerne, but also in the surrounding agricultural areas as expected. Furthermore, as expected, some aquatic plants were detected in subsamples from *A. penelope* and *A. anser*. Though considering the complications that arise with the use of eDNA metabarcoding, this study indicates, that a comprehensive examination of food items ingested by waterfowl can be done. In this investigation, the need for enhanced taxonomical resolution may not be essential, as the combination of morphological analysis and DNA metabarcoding can yield adequate information to address pertinent ecological inquiries.

The overlaps in observed richness (Table 4) for the microscopically analysed subsamples were not to be expected if there had been sufficient sample size, so a lack of samples analysed might be the explanation for what is observed. The observed overlap could also be the product of an insufficient reference list for comparison (Table A1). The cumulative curves do not tend to flatten to a particular degree, and the 95%-confidence interval is rather large, signifying a large spread in the results calculated (Figure 1, Figure 2, Figure 3 and Figure 4). In addition, the cumulative number of taxa in the samples did not reach the theoretical maximum, the calculated Chao estimate (Table 4), which all emphasises the possibility of a lack of sample size or accuracy in species determination.

## 5. Conclusions

This study indicates that a comprehensive examination of the diet of waterfowl can be done by combining the two non-invasive methods of microscopy and eDNA metabarcoding, and the methods can yield adequate information to address pertinent ecological inquiries.

## Figures and Tables

**Table 1:** Schematic overview of microscopic analysis results of the plant species found for *A. penelope*, *A. anser*, *A. brachyrhynchus* and *B. leucopsis*. The frequency of occurrence and the corresponding percentage calculated from the frequency of occurrence value are listed for each plant species found for each waterfowl species.

Plant Species	*A. penelope*		*A. anser*		*A. brachyrhynchus*		*B. leucopsis*	
	FO	%	FO	%	FO	%	FO	%
*Agrostis capillaris*			7	9.86	8	10.96	6	7.32
*Agrostis stolonifera*	21	16.41	8	11.27	12	16.44	12	14.63
*Armeria maritima*			1	1.41	2	2.74		
*Carex nigra*	1	0.78					2	2.44
*Cynosurus cristatus*	1	0.78	5	7.04	2	2.74	6	7.32
*Festuca rubra*	19	14.84	10	14.08	9	12.33	15	18.29
*Glaux maritima*	50	39.06	11	15.49	5	6.85	10	12.20
*Holcus lanatus*	9	7.03	16	22.54	15	20.55	18	21.95
*Hordeum vulgare*			2	2.82				
*Juncus articulatus*	5	3.91			1	1.37	2	2.44
*Leontodon autumnalis*	12	9.38	3	4.23	5	6.85	2	2.44
*Phragmites australis*	2	1.56						
*Plantago maritima*	1	0.78	1	1.41			1	1.22
*Potentilla anserina*	1	0.78	1	1.41	7	9.59		
*Puccinellia maritima*							7	8.54
*Trifolium pratense*	3	2.34	4	5.63	5	6.85	1	1.22
*Triglochin maritima*			2	2.82	1	1.37		
*Triticum aestivum*	2	1.56			1	1.37		
*Zostera marina*	1	0.78						
Total FO	128	100	71	100	73	100	82	100
Total number of samples	55		20		20		25	

**Table 2:** Schematic overview of results from eDNA analysis of the droppings of *A. penelope*, *A. anser*, *A. brachyrhynchus*, and *B. leucopsis*. The number indicates the number of subsamples in which the plant order, family or species have been found for each waterfowl species. Species that are marked (*), are either rare or unknown within the study area.

			*A. penelope*	*A. anser*	*A. brachyrhynchus*	*B. leucopsis*
Order	Family	Species	Number of Samples			
Asparagales				1	1	
Asterales	Asteraceae	*Achillea* spp.	1	1	1	
Asterales	Asteraceae	*Bellis perennis*			1	
Asterales	Asteraceae	*Cirsium* spp.			2	
Asterales	Asteraceae	*Leontodon autumnalis*	7	7	12	15
Asterales	Asteraceae	*Leontodon* spp.				1
Capparales	Brassicaceae	*Brassica napus*		3		1
Capparales	Brassicaceae	*Cardamine hirsuta*		1		
Capparales	Brassicaceae	*Cardamine impatiens* *			1	7
Caryophyllales	Caryophyllaceae	*Cerastium fontanum*	1		2	
Caryophyllales	Caryophyllaceae	*Lychnis flos-cuculi*				1
Caryophyllales	Caryophyllaceae	*Sagina* spp.		1	1	3
Caryophyllales	Caryophyllaceae	*Spergularia media*	1		1	
Cyperales	Cyperaceae	*Carex lasiocarpa*		1	2	10
Cyperales	Cyperaceae	*Eleocharis uniglumis*		3	1	1
Cyperales	Cyperaceae	*Schoenoplectus tabernaemontani*		3		
Cyperales	Cyperaceae	*Schoenus nigricans* *	1	3	1	
Fagales	Betulaceae	*Betula pendula*		1		
Fabales	Fabaceae	*Trifolium pratense*				2
Fabales	Fabaceae	*Trifolium repens*	16	8	13	13
Fabales	Fabaceae	*Vicia sativa*		1		
Gentianales	Gentianaceae		1			
Gentianales	Rubiaceae	*Galium* spp.	1		1	
Hypnales	Amblystegiaceae	*Drepanocladus sendtneri* *	3	1	1	1
Hypnales	Brachytheciaceae	*Kindbergia praelonga*	3	2	2	2
Juncales	Juncaceae	*Juncus bufonius*	4	4	5	1
Juncales	Juncaceae	*Juncus bulbosus*		2	2	4
Juncales	Juncaceae	*Juncus conglomeratus*	1		1	
Juncales	Juncaceae	*Juncus gerardii*	17	9	12	11
Papaverales	Papaveraceae	*Meconopsis* spp. *			1	
Pinales	Pinaceae	*Pinus mugo*	1	2	2	2
Poales	Poaceae	*Agrostis capillaris*	2	2	7	10
Poales	Poaceae	*Alopecurus geniculatus*	2	7	15	18
Poales	Poaceae	*Anthoxanthum* spp.		1		1
Poales	Poaceae	*Briza* spp.		5	2	7
Poales	Poaceae	*Anisantha sterilis* *			1	
Poales	Poaceae	*Calamagrostis arundinacea* *	1			
Poales	Poaceae	*Catabrosa aquatica*	1			
Poales	Poaceae	*Cynosurus cristatus*		2	7	5
Poales	Poaceae	*Festuca arundinacea*	4	9	19	19
Poales	Poaceae	*Festuca* spp.	11	13	17	20
Poales	Poaceae	*Glyceria declinata* *		1		4
Poales	Poaceae	*Holcus lanatus*		3		1
Poales	Poaceae	*Hordeum vulgare*		6	2	
Poales	Poaceae	*Phragmites australis*	3	4	1	
Poales	Poaceae	*Poa pratensis*	1	3	3	9
Poales	Poaceae	*Poa supina* *		1	5	3
Poales	Poaceae	*Poa trivialis*	18	13	18	18
Poales	Poaceae	*Triticum aestivum*		1	1	1
Polygonales	Polygonaceae	*Rumex crispus*			1	
Primulales	Myrsinaceae	*Glaux maritima*	15	4	2	1
Ranunculales	Ranunculaceae	*Batrachium* spp.	1			
Ranunculales	Ranunculaceae	*Myosurus minimus* *			1	
Ranunculales	Ranunculaceae	*Ranunculus repens*		1	6	4
Rosales	Rosaceae	*Potentilla anserina*	17	10	9	13
Rosales	Rosaceae	*Potentilla* spp.				1
Scrophulariales	Lentibulariaceae	*Utricularia australis* *	1			
Scrophulariales	Orobanchaceae		3	1	2	
Scrophulariales	Plantaginaceae	*Plantago major*			2	1
Scrophulariales	Plantaginaceae	*Plantago maritima*	12	5	4	2
Solanales	Boraginaceae	*Myosotis arvensis*			1	
Solanales	Convolvulaceae	*Cuscuta* spp. *	1			
Solanales	Solanaceae	*Solanum* spp.		1		
Zosterales	Juncaginaceae	*Triglochin maritima*		1		
Zosterales	Potamogetonaceae	*Potamogeton perfoliatus* *	2	2		
Total number of samples			21	19	19	20

**Table 3:** Schematic overview of the overall results on species level from microscopic and eDNA analysis of the droppings of all four bird species. Observation of a plant species by microscopic- and eDNA analysis is marked by M and E, respectively.

Species	*A. penelope*	*A. anser*	*A. brachyrhynchus*	*B. leucopsis*
*Achillea* spp.	E	E	E	
*Agrostis capillaris*	E	M/E	M/E	M/E
*Agrostis stolonifera*	M	M	M	M
*Alopecurus geniculatus*	E	E	E	E
*Anisantha* *sterilis*			E	
*Anthoxanthum* spp.		E		E
*Armeria maritima*		M	M	
*Batrachium* spp.	E			
*Bellis perennis*			E	
*Betula pendula*		E		
*Brassica napus*		E		E
*Briza* spp.		E	E	E
*Calamagrostis arundinacea*	E			
*Cardamine hirsuta*		E		
*Cardamine impatiens*			E	E
*Carex lasiocarpa*		E	E	E
*Carex nigra*	M			M
*Catabrosa aquatica*	E			
*Cerastium fontanum*	E		E	
*Cirsium* spp.			E	
*Cuscuta* spp.	E			
*Cynosurus cristatus*	M	M/E	M/E	M/E
*Drepanocladus sendtneri*	E	E	E	E
*Eleocharis uniglumis*		E	E	E
*Festuca arundinacea*	E	E	E	E
*Festuca rubra*	M	M	M	M
*Festuca* spp.	E	E	E	E
*Galium* spp.	E		E	
*Glaux maritima*	M/E	M/E	M/E	M/E
*Glyceria declinata*		E		E
*Holcus lanatus*	M	M/E	M	M/E
*Hordeum vulgare*		M/E	E	
*Juncus articulatus*	M		M	M
*Juncus bufonius*	E	E	E	E
*Juncus bulbosus*		E	E	E
*Juncus conglomeratus*	E		E	
*Juncus gerardii*	E	E	E	E
*Kindbergia praelonga*	E	E	E	E
*Leontodon autumnalis*	M/E	M/E	M/E	M/E
*Leontodon* spp.				E
*Lychnis flos-cuculi*				E
*Meconopsis* spp.			E	
*Myosotis arvensis*			E	
*Myosurus minimus*			E	
*Phragmites australis*	M/E	E	E	
*Pinus mugo*	E	E	E	E
*Plantago major*			E	E
*Plantago maritima*	M/E	M/E	E	M/E
*Poa pratensis*	E	E	E	E
*Poa supina*		E	E	E
*Poa trivialis*	E	E	E	E
*Potamogeton perfoliatus*	E	E		
*Potentilla anserina*	M/E	M/E	M/E	E
*Potentilla* spp.				E
*Puccinellia maritima*				M
*Ranunculus repens*		E	E	E
*Rumex crispus*			E	
*Sagina* spp.		E	E	E
*Schoenoplectus tabernaemontani*		E		
*Schoenus nigricans*	E	E	E	
*Solanum* spp.		E		
*Spergularia media*	E		E	
*Trifolium pratense*	M	M	M	M/E
*Trifolium repens*	E	E	E	E
*Triglochin maritima*		M/E	M	
*Triticum aestivum*	M	E	M/E	E
*Utricularia australis*	E			
*Vicia sativa*		E		
*Zostera marina*	M			

**Table 4:** Schematic overview of the observed richness and the estimated richness for both eDNA analysis and microscopy. The richness is shown for each taxonomic level and for each of the bird species.

	*A. penelope*		*A. anser*		*A. brachyrhynchus*		*B. leucopsis*	
	Observed	Estimated	Observed	Estimated	Observed	Estimated	Observed	Estimated
Microscopic								
Order	9	14.89	8	12.28	8	9.90	7	7.64
Family	9	14.89	8	12.28	8	9.90	7	7.64
Genus	14	20.14	12	14.14	12	14.14	11	11.64
Species	14	20.14	13	15.14	13	15.14	12	12.64
eDNA								
Order	15	17.54	17	28.84	18	26.53	13	13.24
Family	19	30.67	20	30.11	20	25.80	14	14.95
Genus	27	49.86	37	58.32	37	52.21	26	41.20
Species	30	61.11	41	61.21	43	56.95	33	42.50

## Data Availability

The data presented in this study are available on request from the corresponding author.

## Appendix A

**Table A1:** Overview of plants used for reference list, in regards to comparison with the microscopic analysis. The table contains the taxonomic levels order, family, genus and species, for each of the collected and used plants.

Order	Family	Species
*Asterales*	*Asteraceae*	*Aster tripolium*
*Asterales*	*Asteraceae*	*Leontodon autumnalis*
*Caryophyllales*	*Caryophyllaceae*	*Spergularia marina*
*Cyperales*	*Cyperaceae*	*Carex nigra*
*Fabales*	*Fabaceae*	*Trifolium pratense*
*Juncales*	*Juncaceae*	*Juncus articulatus*
*Plumbaginales*	*Plumbaginaceae*	*Armeria maritima*
*Poales*	*Poaceae*	*Agrostis capillaris*
*Poales*	*Poaceae*	*Agrostis stolonifera*
*Poales*	*Poaceae*	*Cynosurus cristatus*
*Poales*	*Poaceae*	*Festuca rubra*
*Poales*	*Poaceae*	*Holcus lanatus*
*Poales*	*Poaceae*	*Hordeum vulgare*
*Poales*	*Poaceae*	*Phragmites australis*
*Poales*	*Poaceae*	*Puccinellia maritima*
*Poales*	*Poaceae*	*Triticum aestivum*
*Poales*	*Poaceae*	*Zea mays*
*Primulales*	*Myrsinaceae*	*Glaux maritima*
*Rosales*	*Rosaceae*	*Potentilla* *anserina*
*Scrophulariales*	*Plantaginaceae*	*Plantago maritima*
*Zosterales*	*Juncaginaceae*	*Triglochin maritima*
*Zosterales*	*Zosteraceae*	*Zostera Marina*

**Table A2:** Schematic overview of the number of reads obtained per sample as well as the total for each waterfowl species and the grand total. The reads have been obtained by using a trnL c/d primerset, 12S primers and archaea/bakteria/eukaryotic primers.

*A. penelope* Sample nr.	Number of Reads	*A. anser* Sample nr.	Number of Reads	*A. brachyrhynchus* Sample nr.	Number of Reads	*B. leucopsis* Sample nr.	Number of Reads
1	5938	1	5436	1	7552	1	8312
2	7747	2	6110	2	6321	2	8207
3	7102	3	6734	3	5558	3	5533
4	8978	4	8339	4	5904	4	3593
5	4195	5	8518	5	6795	5	9772
6	8819	6	7614	6	7058	6	4377
7	10,363	7	7278	7	6740	7	5175
8	5426	8	3693	8	6380	8	4060
9	8027	9	7585	9	6986	9	4782
10	4881	10	7339	10	7726	10	6401
11	7122	11	12,271	11	11,804	11	7402
12	8304	12	7980	12	5545	12	5937
13	5446	13	8029	13	7806	13	9759
14	446	14	5607	14	14,973	14	5455
15	9189	15	11,932	15	6511	15	8557
16	11,112	16	9127	16	7053	16	7477
17	8069	17	20,479	17	8304	17	6523
18	7265	18	13,648	18	5180	18	8194
19	10,229	19	13,220	19	9967	19	10,154
20	9959					20	7524
21	11,144						
Total number of reads	159,761		170,939		144,163		137,194
Grand total							612,057
